# Clinical Efficacy of Ultrasound Cycloplasty for Glaucoma Patients and Pathological Observation on the Ciliary Body in Rabbit Models

**DOI:** 10.1155/joph/1279697

**Published:** 2026-03-30

**Authors:** Liukun Shi, Yufeng Liu, Rui Wang, Wencui Shen, Jin Yang

**Affiliations:** ^1^ Tianjin Eye Hospital, Nankai University Affiliated Tianjin Eye Hospital, Tianjin Key Lab of Ophthalmology and Visual Science, Tianjin Eye Institute, Tianjin, China, oio.cn

**Keywords:** intraocular pressure, refractory glaucoma, ultrasound cycloplasty

## Abstract

**Purpose:**

To evaluate the efficacy and safety of ultrasound cycloplasty (UCP) for glaucoma patients and the ciliary body in rabbit models.

**Methods:**

A prospective case series clinical trial and a animal experiment were carried out separately. For human subjects, 55 patients (55 eyes) with refractory glaucoma underwent UCP. The preoperative and postoperative intraocular pressure (IOP), number of antiglaucoma medications, relevant parameters for measuring the ciliary body after UCP, ocular pain grade, and rate of surgical complications were recorded. For animal subjects, eighteen pigmented rabbits were subjected to UCP, and histological changes were observed under light microscopy and transmission electron microscopy 3 months after surgery.

**Results:**

The mean IOP was 45.05 ± 12.29 mmHg before surgery and 23.30 ± 10.00 mmHg 1 year after surgery, the mean number of antiglaucoma medications used was 3 (2, 3) before surgery and 1 (1, 2) 1 year after surgery, and The Visual Analog Scale score was 5 (4, 6) before surgery and 1(1, 2) 1 year after surgery (both *p* < 0.05). Ultrasound biomicroscopy revealed that the height of the ciliary processes (T value) at 6:00 and 12:00 had increased on Postoperative Day 1, recovered at 1 month after surgery, and decreased at 3 months after surgery for 20 patients. In the animal models, light microscopy revealed that part of the nonpigmented epithelium of the ciliary body had disappeared, the arrangement of the pigmented epithelium was disordered, and the stroma was edematous. Transmission electron microscopy revealed that the mitochondria were damaged and swollen and that the crista structure had dissolved and disappeared.

**Conclusions:**

UCP is effective and safe for refractory glaucoma and requires a long follow‐up time. It can effectively decrease IOP in glaucoma patients, reduce number of antiglaucoma medications, relief the pain, and has fewer complications.

**Trial Registration:** Chinese Clinical Trial Register (ChiCTR): ChiCTR2500114521

## 1. Background

Glaucoma is a leading, irreversible blinding eye disease characterized by optic nerve damage, visual field defects, and an elevated intraocular pressure (IOP). A high IOP is still the most important risk factor for glaucoma progression and vision loss [1]. The goal of the traditional treatment regimen (medication, laser, or surgery) is to achieve a balance between aqueous humor inflow and outflow by decreasing production and/or increasing drainage. As a result, the IOP can be controlled. When topical medications or laser therapy fail to control IOP, incisional surgery should be considered. Traditional surgeries for glaucoma include glaucoma filtration surgery and ciliary body surgery. The probability of postoperative complications should be considered before surgery. Traditional glaucoma filtering surgery involves a large incision and is likely to cause bleeding‐related complications. Traditional ciliary body surgery can cause serious complications, such as chronic hypotony, phthisis, chronic inflammation, macular edema, and retinal detachment because of the nonselective action of this cyclodestructive procedure over the target tissues and the arbitrary dose‒effect relationship [2]. Glaucoma that does not respond to treatment with conventional surgery is called refractory glaucoma, and glaucoma that fails to respond to treatment with multiple filtering operations progresses to neovascular glaucoma (NVG). Glaucoma may even develop following vitrectomy or present secondary to uveitis or trauma, thus cyclodestructive procedures are often used to reduce the IOP and relieve ocular pain in these patients [3]. However, it still has serious complications, such as eyesight loss and even eyeball atrophy.

In the 1980s, high‐intensity–focused ultrasound (HIFU) was introduced as a method to selectively target the ciliary body. By concentrating ultrasound beams through optically opaque ocular media, HIFU achieves controlled energy absorption while minimizing damage to adjacent structures [4]. However, this technique has been abandoned because of its complexity, the long time required in the operating room, the bulky probe that is often inaccurately positioned, and the larger focal zone of treatment, all of which can increase the risk of tissue damage and other complications [4].

Ultrasound cycloplasty (UCP) was developed to achieve selective and controlled thermal effects on the ciliary body via HIFU. As a new type of noninvasive annular destruction surgery, UCP effectively reduces IOP through selective transscleral coagulation necrosis of the ciliary body epithelium and increases suprachoroidal and transsscleral aqueous humor outflow [5]. Focused ultrasound is advantageous because energy can be concentrated within a targeted tissue range, thereby minimizing collateral damage [6]. Initial reports revealed that UCP reduced IOP in patients with various types of glaucoma [7]. According to the 2020 Chinese Glaucoma Guidelines, UCP is a key method of treating NVG [8]. However, the relevant parameters for measuring the ciliary body during ultrasound biomicroscopy (UBM) and the histopathological changes in the ciliary body after UCP remain largely undetermined. The purpose of this study was to observe the clinical efficacy of UCP in patients with refractory glaucoma and to observe histological changes after surgery in animal models.

## 2. Methods

### 2.1. Human Subjects

In this prospective study, we included a cohort of 55 patients (55 eyes) with refractory glaucoma who underwent UCP at Tianjin Eye Hospital from July 2021 to May 2024. The study was approved by the Ethics Committee of Tianjin Eye Hospital (Scientific Research Review No. 202110), and all procedures were performed in adherence to the tenets of the Declaration of Helsinki, and all patients signed a single informed consent form and were followed after the study.

The inclusion criteria were as follows: (1) patients who were aged 24 to 90 years and (2) diagnosed with glaucoma on the basis of an IOP ≥ 30 mmHg (1 mmHg = 0.133 kPa) and receiving the maximum dose of antiglaucoma medication with or without a history of previous glaucoma surgery. The exclusion criteria were as follows: (1) patients with a recent history of active inflammation of the eye, (2) patients who failed to complete the scheduled follow‐ups for various reasons, (3) patients whose axial length (AL) was < 18 mm or > 36.5 mm or whose white‐to‐white corneal diameter (WTW) was > 13 mm, so there was no suitable probe model for surgery, (4) patients who were pregnant, and (5) patients with a history of either refractory surgery, retinal detachment, or an ocular tumor.

The preoperative evaluation included best corrected visual acuity (BCVA), slit‐lamp microscopy (BM900, SwissHager) and IOP measurement, primary disease classification, and UBM (MD‐300 L, TianjinMida) with a Lenstar optical low‐coherence reflectance measuring instrument (LS900, Haag‐Streit). BCVA was converted to the logarithm of the minimum angle of resolution (LogMAR), and IOP was measured with a Goldmann tonometer (AT 900, Haag‐Strsit AG, Switzerland, Bern), except in patients with corneal edema due to a high IOP, in whom an Icare handheld rebound tonometer (TA01i, Finland Aike Company, Finland, Helsinki) was used.

### 2.2. Surgery

Before treatment, the biometrics of the WTW and AL were measured to select the appropriate probe size (11, 12, or 13 mm). UCP was performed with a second‐generation Eyeop1 probe (Eye Tech Care, France). The standard parameters were selected according to the preoperative IOP (6 sectors when the IOP was 21–30 mmHg, 8 sectors when the IOP was 31–40 mmHg, 10 sectors when the IOP was greater than 40 mmHg, a frequency of 21 MHz, a duration of 8 s, and an interval of 20 s). All patients were treated by the same physician (JY). All procedures were performed under retrobulbar anesthesia. First, a vacuum test was performed, the positioning cone was aligned and fixed on the ocular surface, and the negative pressure was stable. The probe was inserted into the positioning cone, which was then filled with balance salt solution to allow for ultrasound transmission, and the start button was pressed to automatically start the treatment procedure [9]. At the end of the procedure, retrobulbar injection of 40 mg of methylprednisolone was used to reduce the intraocular inflammatory response [10]. Postoperatively, levofloxacin, prednisolone acetate, and sodium hyaluronate eye drops were applied 4 times a day, and tobramycin dexamethasone eye ointment was applied once a night for 2 months or until the inflammation of the anterior segment disappeared. Antiglaucoma drops were resumed, but the dose was changed according to the follow‐up IOP.

### 2.3. Follow‐Up

Patients returned for follow‐up visits on the first postoperative day, at 1 week and 2 weeks after surgery, at 1 month and 3 months after surgery, and 6 months and 1 year after surgery. BCVA and IOP were recorded. Slit‐lamp microscopy, ocular pain grade, related parameters of the ciliary body, number of antiglaucoma medications, and intraoperative and postoperative complications were recorded. The visual analog scale (VAS) was used to assess the subjective feeling of pain (a score of 0 indicates no pain, 1∼3 indicates mild pain, 4∼6 indicates moderate pain, and 7∼10 indicates severe pain) [11].

For 20 of the patients, we measured the distance from the ciliary process to the attachment point of the iris root and the height of the ciliary process during UBM at 3, 6, 9, and 12 points at preoperation, 1 day, 1 month, and 3 months after surgery. The height of the ciliary process refers to the vertical distance between the highest point of the ciliary process and the inner surface, also known as the T value.

In a previous study, the researchers suggested that complete success was defined as an IOP controlled within 6–21 mmHg without antiglaucoma medications, qualified success was defined as an IOP controlled within 6–21 mmHg with antiglaucoma medications, and failure was defined as an IOP > 21 mmHg at 2 consecutive follow‐up visits even with the maximum tolerated antiglaucoma medication, persistent hypotony (IOP < 5 mmHg) at 2 visits, loss of light perception, severe complications, or the need for additional glaucoma surgery to reduce the IOP, including a repeat UCP [12]. Success rate of surgical treatment = (number of completely successful cases + number of conditionally successful cases)/total number of cases × 100%.

### 2.4. Animal Subjects

Eighteen healthy adult pigmented rabbits (provided by the Experimental Animal Center of Tianjin Nankai Hospital) were subjected to enucleation for pathological observation (light microscopy and transmission electron microscopy) 3 months after UCP. In each rabbit, Ketamine hydrochloride (20 mg/kg) and xylazine hydrochloride (3 mg/kg) was injected into the thigh muscle to induce anesthesia, and the eyes were treated with UCP. Postoperative care for rabbits involves close monitoring of vital signs, pain management, and anti‐inflammatory treatment; providing a suitable environment and nutrition, and restricting activity to ensure proper recovery and compliance with ethical standards. The study was approved by the Ethics Committee for Experimental Animals (No: NKYY‐DFL‐2020) and was conducted in accordance with animal protection, animal welfare, and ethical principles.

The surgically excised eyeballs were immediately immersed in a solution of 10% formalin fixative at room temperature for 24 h, and these samples were then dehydrated with different concentrations of alcohol (from 70% to 100%) and made transparent via two washes in dimethylbenzene. After the tissues were embedded in paraffin, 5 μm‐thick sections were cut, mounted, and allowed to dry overnight. The samples were subsequently stained with haematoxylin and eosin and observed with a light microscope (Leica 400B, Leica Company, Germany, Solms).

The ciliary bodies of the other eyes were immersed in 4% glutaraldehyde and 1% osmic acid solutions at 4°C for 4 and 1 h, respectively, and these specimens were then washed with PBS powder and dehydrated through a gradient of acetone solutions. After the tissues were embedded in 812 resin, the samples were cut into 60‐nm‐thick sections and stained with lead citrate and uranyl acetate. Transmission electron microscopy (JEM‐1230, JEOL Company, Japan, Tokyo) was used to view these sections.

### 2.5. Statistical Analysis

Statistical analyses were performed via SPSS 31.0 statistical software (IBM, USA). The data on IOP and ciliary body–related parameters were confirmed by the Shapiro–Wilk test and were normally distributed. Therefore, these results are presented as the mean ± standard deviation (SD). The data on the number of antiglaucoma drugs used and the VAS score were not normally distributed. Therefore, these results are expressed as M (Q1, Q3). A paired sample *t* test was used to assess differences between the IOP and ciliary body–related parameters at different time points. The number of antiglaucoma medications used and the VAS score were compared across groups via the Wilcoxon rank sum test. *p* values lower than 0.05 indicated statistical significance.

## 3. Results

This study included 27 males (27 eyes) and 28 females (28 eyes) ranging in age from 24 to 90 (45 ± 13) years. Thirty‐three patients (33 eyes) completed the entire follow‐up. Among all the recruited eyes, 13 eyes had primary angle‐closure glaucoma (PACG), 16 eyes had NVG, and 26 eyes had other types of secondary glaucoma. Among all 55 eyes, 37 eyes never underwent glaucoma surgery, and 18 eyes previously underwent glaucoma surgery. The baseline IOP was 45.05 ± 12.29 mmHg. The characteristics and ocular history of the patients are shown in Table S1.

Data at the 7 follow‐up visits were collected (Table S2). The mean IOP decreased significantly from 45.05 ± 12.29 to 27.02 ± 9.47, 21.29 ± 8.64, 20.48 ± 7.43, 21.98 ± 6.65, 22.33 ± 9.83, and 23.3 ± 10.0 mmHg at the various follow‐up points (both *p* < 0.05). The number of antiglaucoma medications used decreased from 3 (2, 3) to 1 (1, 2), and the mean VAS score decreased from 5 (4, 6) to 1 (1, 2), which was statistically significant (both *p* < 0.05).

The successful operation rates were 70.90%, 65.45%, 59.26%, 58.82%, 53.84%, and 48.48%, at 1 day, 1 week, 1 month, 3 months, 6 months, and 1 year, respectively. The highest success rate is Day 1, and gradually decreased over times. In the NVG group, the success rate ranged from 16.67% to 68.75%, while that in the PACG group was 46.15% to 71.42%. In the Surgery history group, the success rate ranged from 53.84% to 77.78%, while that in the Nonsurgery history group was 45.0% to 67.56% (Table S3).

The IOP reduction rates were recorded at each follow‐up visit (Tables S4 and S5). The IOP reduction rates in the NVG group and PACG group were 40.63% versus 37.61% at 1 day, 52.33% versus 50.94% at 1 week, 51.50% versus 50.59% at 1 month, 49.31% versus 47.39% at 3 months, 40% versus 55.87% at 6 months, and 28.76% versus 58.04% at 1 year, respectively. The IOP reduction rates in the previous surgical history group and no previous surgical history group were 38.67% versus 40.71% at 1 day, 48.88% versus 54.59% at 1 week, 62.12% versus 51.12% at 1 month, 53.43% versus 40.24% at 3 months, 57.77% versus 47.46% at 6 months and 54.36% versus 44.37% at 1 year, respectively.

Ciliary body–related parameters of 20 patients were collected during UBM (Table S6). We found that the height of the ciliary processes (T value) at 6:00 and 12:00 had increased on Postoperative Day 1, had recovered at 1 month after surgery, and had decreased at 3 months after surgery. The change in the T value on Postoperative Day 1 was statistically significant (*p* < 0.05). There was no statistically significant difference in the distance from the ciliary process to the attachment point of the iris root at various follow‐up points (*p* > 0.05). In addition, the UBM images revealed ciliary body choroidal detachment after surgery (Figure S1).

Five patients (9.09%) reported severe intraoperative pain, which resolved postoperatively. Common complications included conjunctival hyperemia, corneal edema, scleral impressions, hypotony, conjunctival edema, hyphema, corneal degeneration, and posterior capsular opacity, most of which had disappeared by the last postoperative follow‐up (Figure S2). No patients experienced ocular atrophy, endophthalmitis or other serious complications.

### 3.1. Animal Subjects’ Outcomes

Under light microscopy, the results demonstrated that some nonpigmented epithelium of the ciliary process fell off and disappeared and that the pigmented epithelium was disordered. The ciliary stroma was edematous; the adjacent tissues around the ciliary process were not diseased, and the capillaries were not involved. There was no obvious damage to the peripheral choroid or retina (Figure S3).

Under transmission electron microscopy, the data indicated that the mitochondria in the nonpigmented epithelial cells were damaged, the mitochondria was swollen, and the cristae structure had dissolved and disappeared; part of the Golgi apparatus was swollen, and the cavities were significantly dilated; there was no damage to the vascular endothelial cells, and the endothelium was smooth (Figure S4).

## 4. Discussion

Refractory glaucoma is a complex type of glaucoma that usually has a poor prognosis, and its treatment is more complicated. Traditional medicine, lasers, and filtering surgery sometimes cannot effectively control the IOP. A cyclodestructive procedure is performed in patients, but obvious eyeball pain, severe damage to eye tissue, and severe postoperative complications often occur due to the low‐temperature effect [3]. In the 1980s, HIFU, which involves the use of ultrasound as a medium to transmit energy and therefore create a thermal effect that destroys the target organ without affecting the tissues around the target organ, was used to treat glaucoma [4]. However, HIFU has been abandoned because it is a complex procedure that is time‐consuming and more suitable for large treatment areas. Therefore, UCP, as a new minimally invasive surgery, has been increasingly performed in clinical practice. UCP is a new type of incisionless destructive procedure that directly reduces aqueous humor production by destroying ciliary achromatic epithelial cells, and owing to posttreatment ciliary contraction, the potential space between the ciliary body and the sclera increases, resulting in increased aqueous humor outflow through the uveoscleral pathway [13]. Selective vascular embolic necrosis of the ciliary body and ciliary process was found in UCP‐treated rabbits [14], and in patients with refractory glaucoma after UCP, increased intrascleral hyporeflectance space and increased conjunctival microcapsules in the ultrasound penetration zone were found, suggesting increased aqueous humor outflow through the sclera [15]. The circular probe used in EyeOP1 is precisely coupled to the target organ, which avoids damage to adjacent structures, and the patient’s nasal and temporal sides are not treated with ultrasound, thereby avoiding the long ciliary nerve and reducing pain [16]. The whole procedure takes only a few minutes, and the risk of surgery is significantly lower [17]. As a new way to treat glaucoma, UCP effectively lowers IOP in various types of glaucoma patients [18–22].

Our study revealed that the IOP reduction rate was 48% at 1 year after the operation, and the surgical success rate reached 69.7%. Melamed et al. [23] reported that the IOP decreased from 36.4 ± 5.7 to 22.5 ± 10.3 mmHg in 20 patients with refractory glaucoma after UCP surgery, with an IOP reduction rate of 38.19%, which was due to the low baseline IOP in these patients. Wang et al. [24] reported that the surgical success rate was 77.8%, which was slightly higher than that in our study, possibly because of the different disease stages and different types of refractory glaucoma included. Postoperative antiglaucoma medication use was reduced over time, and the VAS score significantly decreased. Compared with previous ciliary cryosurgery and photocoagulation, UCP can effectively and safely control IOP, thus relieving pain, which is the goal of treatment for advanced glaucoma [25, 26]. A study revealed a significant linear relationship between the decrease in IOP at 3 months and the preoperative IOP, which was also confirmed by Giannaccare et al. [27]. Therefore, patients with a higher preoperative IOP would have a greater reduction in IOP after surgery, indicating that UCP may be more effective for patients with a higher IOP. On the other hand, patients with a lower baseline IOP may have a smaller decrease in IOP, thereby reducing the risk of hypotony. In addition, the preoperative IOP is correlated with the VAS score, which may stimulate pain‐sensing nerves after the IOP increases. If the IOP continues to be high, the cornea may become damaged, resulting in worse eye pain [28].

In our study, we compared the efficacy of UCP in different types of patients by analyzing the data of 16 NVG patients and 13 PACG patients. The results indicated that the PACG group experienced a better IOP‐lowering effect than the NVG group did. Wang [24] also compared the efficacy of UCP for NVG and other types of refractory glaucoma, and the degree of IOP reduction in the two groups was 41.30% and 53.50%, indicating that UCP is slightly less effective in treating NVG. Multiple studies have revealed that the success rate of UCP differs for different types of glaucoma [29, 30]. UCP is a better treatment for PACG, possibly because PACG is caused by poor aqueous humor drainage function caused by the closure of the angle; however, the production of aqueous humor is generally normal, so UCP acts on the ciliary body, destroys the ciliary epithelial tissue, causes coagulative necrosis contraction of the ciliary body, and reduces the production of aqueous humor. The reason for the slightly worse treatment effect in patients with secondary refractory glaucoma, such as NVG, may be that the amount of ciliary tissue coagulation and the reduction in aqueous humor production are insufficient.

In our study, we compared the UCP treatment effects between patients who had not undergone surgery (37) and those with a history of surgery (18) and found that patients who had not undergone surgery have a better treatment effect, which was unlike the results of previous studies [31].

Because the probe used in UCP is in direct contact with the patient’s bulbar conjunctiva and the ultrasound energy is focused on the ciliary body, conjunctival injection and anterior chamber reactions are the most common, followed by superficial punctate keratitis, pupillary changes, focal scleral thinning or imprinting, cataract progression, and intraoperative and postoperative pain. Five patients experienced severe intraoperative eye pain; we found that these patients had a long history of high IOP above 40 mmHg (the maximum dosage of glaucoma drugs), and obvious conjunctival congestion before the operation. These conditions would lead to a decrease in the pain threshold of the patients. After 10‐sector treatment was given to the patients, the severe intraoperative pain was largely related to the insufficient anesthetic effect. After increasing the dosage of anesthesia for these patients, the pain was significantly relieved. One patient developed posterior lens capsule opacity, which was considered related to the patient’s previous history of high myopia and multiple glaucoma surgeries, which led to changes in the position and morphology of the ciliary body, so the size of the probe was not suitable. Luo et al. [32] reported a case of peripheral cortical cataract after UCP, which was caused by the use of a small probe during UCP due to inaccurate WTW measurement. Five patients developed corneal edema within 1 to 7 days after the operation, which is more common in the elderly. It may be related to corneal damage caused by long‐term use of glaucoma drugs, local application of corticosteroids after the operation, and contact between the probe and the cornea. It can resolve spontaneously (within 2 weeks) [33]. Three patients experienced temporary hypotony, which might be related to the inflammatory reaction after the surgery. One patient’s condition improved after 1 month postoperation, while the other two patients’ conditions improved after 3 months. For these patients, all IOP‐lowering medications were discontinued, and local application of corticosteroid drugs was used. No serious complications occurred in our study, which is consistent with the findings of many studies [20, 30, 33–37].

UBM allows noninvasive observation of the morphology of the ciliary body and quantitative measurements. In this study, the parameters for measuring the ciliary body via UBM included the distance between the ciliary process and the attachment point of the iris root, which can be used to reflect the location of the ciliary process and the degree of structural changes in the ciliary process. The height of the ciliary process (T value) reflects the size of the ciliary process. We found that the T value at 6:00 and 12:00 had increased on Postoperative Day 1, had recovered at 1 month after surgery, and had decreased at 3 months after surgery. This occurred because the ciliary body thickens on Day 1 after surgery and returns to its normal state at 1 month after surgery, and the ciliary epithelial cells are destroyed at 3 months after treatment. There was no significant change in the T values at the 3:00 and 9:00 positions, as the treatment sector of the probe used in UCP was not placed in the 3:00 and 9:00 positions. There was no significant difference in the distance between the ciliary process and the iris root attachment point at 3 o’, 6 o’, 9 o’ and 12 o’, indicating that UCP affected only the size of the ciliary process and not its position. In addition, there have been cases in which UCP aided in the opening of the uveal‐scleral channel in patients with ciliary choroidal detachment.

At present, UCP can significantly reduce IOP, reduce the use of antiglaucoma drugs, and decrease the time of filtration surgery for all types of glaucoma, but a few pathological observations related to UCP in the ciliary body have been reported. We conducted relevant experiments to observe the pathological changes following UCP and further validated the mechanism by which UCP lowers IOP. In the animal models, we found that the nonpigmented epithelium of the ciliary body was absent, the pigment epithelium was disorganized, and the stroma was edematous at 3 months after treatment. Transmission electron microscopy revealed that the mitochondria were damaged and swollen and that the crest structure had dissolved and disappeared. A portion of mitophagy formed autophagosomes; partial Golgi swelling and marked enlargement of the cavity further confirmed the destruction of the ciliary process after UCP. In our animal experiments, we found that UCP causes coagulative necrosis of the ciliary epithelium, but no damage to the surrounding scleral tissue was observed. This is consistent with previous studies on the postoperative aqueous humor dynamics of UCP, which indicated that UCP altered the flow rate of aqueous humor, suggesting that the mechanism by which UCP lowers IOP mainly results from reducing ciliary secretion. However, due to the anatomical differences between rabbit eyes and human eyes, the mechanism by which UCP lowers IOP still requires further investigation [31, 38].

At present, the main reasons for the resurgence of IOP after UCP are as follows: (1) It is related to the pathogenesis of glaucoma, such as NVG; (2) failure to select the appropriate sector according to the patient’s actual situation; (3) early failure (within 6 months) may be due to an insufficient amount of coagulated ciliary tissue during surgery; (4) late failure (more than 6 months) may reflect re‐epithelialization of the ciliary process, and its function is gradually restored. However, only a small sample of patients was included in our study, and the follow‐up time was short. Thus, future research is needed to further explore the relationship between ciliary body re‐epithelialization and increased IOP [27].

Due to the anatomical differences between rabbit eyes and human eyes, as well as the fact that the animal model used in this study is a normal IOP rabbit model, the results of the animal experiments still need to be further validated by establishing a glaucoma animal model to better elucidate the mechanism by which UCP lowers IOP. Due to the relatively short follow‐up period of this experiment, the relationship between the frequency and interval of repeated UCP treatment and the treatment effectiveness was not thoroughly explored. In the future, a larger sample size and longer follow‐up period will be adopted to investigate the effectiveness of repeated UCP treatment.

## 5. Conclusions

UCP can damage ciliary epithelial cells without disrupting the blood‒water barrier. It can effectively and safely control the IOP in refractory glaucoma patients, relieve eye pain, and reduce complication rates. In addition, UBM allows noninvasive examination of the shape of the ciliary body after UCP, which is of great clinical significance. Studies with longer follow‐up periods are needed to further confirm the long‐term efficacy and safety of UCP. Owing to the poor visual acuity of the subjects included in this study, the effectiveness and safety of UCP in patients with a high IOP with good visual acuity need to be further explored in the future, and the treatment scope will be expanded to investigate whether UCP causes peripheral visual field defects.

**Nomenclature**
UCP: Ultrasound cycloplasty
IOP: Intraocular pressure
NVG: Neovascular glaucoma
HIFU: High‐intensity–focused ultrasound
WTW: White‐to‐white corneal diameter
BCVA: Best corrected visual acuity
UBM: Ultrasound biomicroscopy the
LogMAR: Logarithm of the minimum angle of resolution
VAS: Visual Analog Scale

## Author Contributions

Liukun Shi: Data collection, analysis, and interpretation; Yufeng Liu: drafting of the manuscript; Rui Wang: histopathological analysis; Wencui Shen: statistical analysis; Jin Yang: study design, case selection, data interpretation, and critical revision of the manuscript. Liukun Shi, Yufeng Liu, and Rui Wang shared first authorship.

## Funding

This study was financially supported by the Key Project of Tianjin Eye Hospital (YKZD1905) and Tianjin Key Medical Discipline Construction (TJYXZDXK‐3‐004A‐3), and the funding provided technical support, a theoretical basis, and a hardware guarantee for the design of the study, the collection, analysis, and interpretation of the data and the writing of the manuscript.

## Disclosure

All authors read and approved the final manuscript.

## Ethics Statement

This study was approved by the Ethics Committee of Tianjin Eye Hospital (Scientific Research Review No. 202110) and the Ethics Committee for Experimental Animals (No: NKYY‐DFL‐2020), and all procedures were performed in accordance with the Declaration of Helsinki. All patients provided voluntary informed consent to participate in the study. Written consent was obtained from the participants.

## Consent

Please see the Ethics Statement.

## Conflicts of Interest

The authors declare no conflicts of interest.

## Supporting Information

Additional supporting information can be found online in the Supporting Information section.

## Supporting information


**Supporting Information 1** Table 1: Patient characteristics and ocular history.

**Supporting Information 2** Table 2: IOP, antiglaucoma medications, and VAS scores.

**Supporting Information 3** Table 3: Success rate in all patients and comparison between NVG and PACG, surgery history and nonsurgery history.

**Supporting Information 4** Table 4: IOP reduction between the NVG group and the PACG group.

**Supporting Information 5** Table 5: IOP reduction between previous surgical history group and no previous surgical history group.

**Supporting Information 6** Table 6: Related parameters of the ciliary body.

**Supporting Information 7** Figure 1: UBM images.

**Supporting Information 8** Figure 2: Images of postoperative complications.

**Supporting Information 9** Figure 3: Light microscopy.

**Supporting Information 10** Figure 4: Transmission electron microscopy.

## Data Availability

The data that support the findings of this study are available from the corresponding author upon reasonable request.

## References

[1] Gordon M. O. , Beiser J. A. , Brandt J. D. et al., The Ocular Hypertension Treatment Study: Baseline Factors that Predict the Onset of Primary open-angle Glaucoma, Archives of Ophthalmology. (2002) 120, no. 714–20, 829–830, 10.1001/archopht.120.6.714, 2-s2.0-0036272299. 12049575

[2] Frezzotti P. , Mittica V. , Martone G. et al., Longterm Followup of Diode Laser Transscleral Cyclophotocoagulation in the Treatment of Refractory Glaucoma, Acta Ophthalmologica. (2010) 88, no. 1, 150–155, 10.1111/j.1755-3768.2008.01354. 19432863

[3] Zhu Y. H. , Liu S. Q. , Wang S. et al., Clinical Study of Transscleral Cyclophotocoagulation for Refractory Glaucoma, Chinese Journal of Practical Ophthalmology. (2015) 33, no. 6, 630–634.

[4] Ishida K. , Update on Results and Complications of Cyclophotocoagulation, Current Opinion in Ophthalmology. (2013) 24, no. 2, 102–110, 10.1097/ICU.0b013e32835d9335, 2-s2.0-84876294117. 23313903

[5] Mastropasqua R. , Agnifili L. , Fasanella V. et al., Uveo-Scleral Outflow Pathways After Ultrasonic Cyclocoagulation in Refractory Glaucoma: an Anterior Segment Optical Coherence Tomography and in Vivo Confocal Study, British Journal of Ophthalmology. (2016) 100, no. 12, 1668–1675, 10.1136/bjophthalmol-2015-308069, 2-s2.0-84959432618. 26883868

[6] Hugo J. , Matonti F. , Beylerian M. , Zanin E. , Aptel F. , and Denis D. , Safety and Efficacy of high-intensity Focused Ultrasound in Severe or Refractory Glaucoma, European Journal of Ophthalmology. (2021) 31, no. 1, 130–137, 10.1177/1120672119874594, 2-s2.0-85074025827. 31550914

[7] Giannaccare G. , Pellegrini M. , Bernabei F. et al., A 2-year Prospective Multicenter Study of Ultrasound Cyclo Plasty for Glaucoma, Scientific Reports. (2021) 11, no. 1, 10.1038/s41598-021-92233-9. PMC8209230 34135447

[8] Glaucoma Group of Ophthalmology Branch of Chinese Medical Association , Ophthalmologist of Chinese Medical Doctor Association Chapter Glaucoma group, Chinese Glaucoma Guidelines. (2020) 56, no. 8, 573–586.

[9] Giannaccare G. , Sebastiani S. , and Campos E. C. , Ultrasound Cyclo Plasty in Eyes with Glaucoma, Journal of Visualized Experiments: JoVE. (2018) 131, 10.3791/56192, 2-s2.0-85041101612. PMC5908701 29443031

[10] Ying J. , Yu S. P. , and Ye L. , Clinical Observation on the Treatment of Intraocular Inflammatory Responses After Combined Surgery of Cornea and Iris with Intracameral Injection of Methylprednisolone, Zhejiang Clinical Medicine. (2015) 17, no. 9, 1541–1542.

[11] Zhou M. , Xu X. , Zhang X. , and Sun X. , Clinical Outcomes of Ahmed Glaucoma Valve Implantation with or Without Intravitreal Bevacizumab Pretreatment for Neovascular Glaucoma: a Systematic Review and Meta-Analysis, Journal of Glaucoma. (2016) 25, no. 7, 551–557, 10.1097/IJG.0000000000000241, 2-s2.0-84978720933. 25719237

[12] Faisal A. , Ahmed A. , Waleed A. , Abdullah A. , and Essam A. , Outcomes and Predictors of Failure of Ultrasound Cyclo Plasty for Primary Open-Angle Glaucoma, Journal of Clinical Medicine. (2022) 11, 10.3390/jcm11226770. PMC9695056 36431247

[13] Aptel F. , Razavi A. , Begle A. et al., Short-And long-term Effects on the Ciliary Bldy and the Aqueous Outflow Pathways of high-inthesity Focused Ultrasound Cyclocoagulation, Ultrasound in Medicine and Biology. (2014) 40, no. 9, 2096–2106, 10.1016/j.ultrasmedbio.2014.04.017, 2-s2.0-84905566031. 24996576

[14] Aptel F. , Charrel T. , Palazzi X. , Chapelon J. Y. , Denis P. , and Lafon C. , Histologic Effects of a New Device for high-intensity Focused Ultrasound Cyclocoagulation, Investigative Ophthalmology & Visual Science. (2010) 51, no. 10, 5092–5098, 10.1167/iovs.09-5135, 2-s2.0-77958127234. 20484580

[15] Roquancount T. , Aptel F. , Rouland J. F. et al., UBM Evaluation of Mechanisms that Drive Intraocular Pressure Decrease After Ultrasound Ciliary Plasty with High Intensity Focused Ultrasound,Towards a New Explanation of the Role of Uveoscleral Pathway Outflow, Acta Ophthalmologic. (2017) 95, no. S259.

[16] Posarelli C. , Covello G. , Bendinelli A. , Fogagnolo P. , Nardi M. , and Figus M. , High Intensity Focused Ultrasound prosedure: The Rise of a New Noninvasive Glaucoma Prosedure and Its Possible Future Applications, Survey of Ophthalmology. (2019) 64, no. 6, 826–834, 10.1016/j.survophthal.2019.05.001, 2-s2.0-85067236108. 31125570

[17] Susan M. C. , Terminology and Guidelines for Glaucoma, Arch De La Sociedad Española De Oftalmología. (2009) 84, no. 10, 543–544.

[18] Almobaraka F. A. , Alrubean A. , Alsharhani W. K. et al., Outcomes of Ultrasound Cycloplasty in Primary Angle Closure Glaucoma Glaucoma, Journal of Glaucoma. (2023) 32, no. 5, 407–413, 10.1097/IJG.0000000000002182. 36795514

[19] Almobaraka F. A. , Alrubean A. , Alsharhani W. K. et al., Two-Year Outcomes of Ultra Sound Cycloplasty as a Safe Procedure in Glaucoma, Semin ophthalmology. (2023) 38, no. 5, 482–489, 10.1080/08820538.2023.2170715. 36762779

[20] MoraisSarmento T. , Figueiredo R. , Garrido J. , Passos I. , Rebelo A. L. , and Candeias A. , Ultrasonic Circular Cyclocoagulation Prospective Safety and Efectiveness Study, International Ophthalmology. (2021) 41, no. 9, 3047–3055, 10.1007/s10792-021-01867-1. 33881668

[21] Wang R. X. , Li N. , and Chen X. Y. , Ultrasoundcyclo:Plastyformoderate Glaucoma: Eighteen, Month Results from a Prospective Study, Frontiers of Medicine. (2022) 9, 10.3389/fmed.2022.1009273. PMC9801481 36590936

[22] Almobaraka F. A. , Alrubean A. , Alsarhani W. K. , Aljenaidel A. , and Osman E. A. , Ultrasound Cycloplasty in Glaucoma: 2-Year Outcomes, Journal of Glaucoma. (2022) 31, no. 10, 834–838, 10.1097/IJG.0000000000002078. 35882024

[23] Melamed S. , Goldenfeld M. , Cotlear D. , Skaat A. , and Moroz I. , High-Intensity Focused Ultrasound Treatment in Refractory Glaucoma Patients: Results at 1 Year of Prospective Clinical Study, European Journal of Ophthalmology. (2015) 25, no. 6, 483–489, 10.5301/ejo.5000620, 2-s2.0-84945941983. 25982212

[24] Wang T. , Wang R. , Su Y. , and Li N. , Ultrasound Cycloplasty for the Management of Refractory Glaucoma in Chinese Patients: a before-after Study, International Ophthalmology. (2021) 41, no. 2, 549–558, 10.1007/s10792-020-01606-y. 33070270

[25] Ruixue W. , Tao W. , and Ning L. , A Comparative Study Between Ultrasound Cycloplasty and Cyclocryotherapy for the Treatment of Neovascular Glaucoma, Journal of Ophthalmology. (2020) 22, 2020–2028, 10.1155/2020/4016536. PMC7001673 32047661

[26] Yu Q. , Liang Y. , Ji F. , and Yuan Z. , Comparison of Ultrasound Cycloplasty and Transscleral Cyclophotocoagulation for Refractory Glaucoma in Chinese Population, BMC Ophthalmology. (2020) 20, no. 1, 10.1186/s12886-020-01655-y. PMC7525941 32993561

[27] Giannaccare G. , Vagge A. , Sebastiani S. et al., Ultrasound Cyclo-Plasty in Patients with Glaucoma: 1-Year Results from a Multicentre Prospective Study, Ophthalmic Research. (2019) 61, no. 3, 137–142, 10.1159/000487953, 2-s2.0-85047135348. 29768281

[28] Chen M. J. , Liu C. J. , Cheng C. Y. , and Lee S. M. , Corneal Status in Primary angle-closure Glaucoma with a History of Acute Attack, Journal of Glaucoma. (2012) 21, no. 1, 12–16, 10.1097/IJG.0b013e3181fc800a, 2-s2.0-84855439208. 21048505

[29] Hu D. , Tu S. , Zuo C. , and Ge J. , Short-Term Observation of Ultrasonic Cycloc-Oagulation in Chinese Patients with End-Stage Refractory Glaucoma: a retrosp-ective Study, Journal of Ophthalmology. (2018) 2018, 1–7, 10.1155/2018/4950318, 2-s2.0-85053703231. PMC6148825 30271627

[30] Denis P. , Aptel F. , Rouland J. F. et al., Cyclocoagulation of the Ciliary Bodies by high-intensity Focused Ultrasound: a 12-month Multicenter Study, Investigative Ophthalmology & Visual Science. (2015) 56, no. 2, 1089–1096, 10.1167/iovs.14-14973, 2-s2.0-84923062782. 25604688

[31] Figus M. , Sartini S. , Covello G. , and Posarelli C. , High-Intensity Focused Ultrasound in the Treatment of Glaucoma: A Narrative Review, Expert Review of Ophthamology. (2021) 16, no. 3, 161–174, 10.1080/17469899.2021.1902309.

[32] Luo J. , Liu Z. , Zhao L. , Zhou Y. , Kong L. , and Sun Y. , Postoperative Complicated Peripheral Cortical Cataract After Ultrasound Cycloplasty: A Case Report, BMC Ophthalmology. (2021) 21, no. 1, 10.1186/s12886-020-01743-z. PMC7789716 33413166

[33] Aptel F. , Dupuy C. , and Rouland J. F. , Treatment of Refractory open-angle Glaucoma Using Ultrasonic Circular Cyclocoagulation: a Prospective Case Series, Current Medical Research and Opinion. (2014) 30, no. 8, 1599–1605, 10.1185/03007995.2014.910509, 2-s2.0-84904900096. 24689838

[34] Giannaccare G. , Vagge A. , Gizzi C. et al., High-Intensity Focused Ultrasound Treatment in Patients with Refractory Glaucoma, Graefes Archive for Clinical and Experimental Ophthalmology. (2017) 255, no. 3, 599–605, 10.1007/s00417-016-3563-z, 2-s2.0-85000977767. 27915382

[35] Rouland J. F. and Aptel F. , Efficacy and Safety of Ultrasound Cycloplasty for Refractory Glaucoma: a 3-Year Study, Journal of Glaucoma. (2021) 30, no. 5, 428–435, 10.1097/IJG.0000000000001796. 33900251

[36] Figus M. , Posarelli C. , Nardi M. et al., Ultrasound Cyclo Plasty for Treatment of Surgery-Naive Open-Angle Glaucoma Patients: a Prospective, Multicenter, 2-Year Follow-Up Trial, Journal of Clinical Medicine. (2021) 10, no. 21, 10.3390/jcm10214982. PMC8584324 34768500

[37] Longfang Z. , Die H. , Jie L. , Yameng L. , Mingyuan L. , and Xiaojing P. , Efficacy and Safety of Single Ultrasound Cyclo-Plasty to Treat Refractory Glaucoma: Results at 1 Year, European Journal of Ophthamology. (2022) 32, no. 1, 268–274, 10.1177/1120672120973605. 33225725

[38] Alaghband P. , Galvis E. , Ramirez A. et al., The Effect of High-Intensity Focused Ultrasound on Aqueous Humor Dynamics in Patients with Glaucoma, Ophthalmology Glaucoma. (2020) 3, no. 2, 122–129, 10.1016/j.ogla. 32672595

