# Supplementary material for: Clinical Efficacy of Ultrasound Cycloplasty for Glaucoma Patients and Pathological Observation on the Ciliary Body in Rabbit Models
Source: J Ophthalmol. 2026 Mar 30;2026:1279697. doi: 10.1155/joph/1279697 (PMC13463128; doi:10.1155/joph/1279697)
Supplement: Supplementary file 1 [file JOPH-2026-1279697-s001.docx]

C

B

A

**Figure.1** Ultrasound biomicroscopy images. **A:**Ciliary body to iris root attachment point.

**B:**Ciliary process’height. **C:** ciliary body choroidal detachment.
