# Supplementary material for: Clinical Efficacy of Ultrasound Cycloplasty for Glaucoma Patients and Pathological Observation on the Ciliary Body in Rabbit Models
Source: J Ophthalmol. 2026 Mar 30;2026:1279697. doi: 10.1155/joph/1279697 (PMC13463128; doi:10.1155/joph/1279697)
Supplement: Supplementary file 2 [file JOPH-2026-1279697-s002.docx]

**Figure. 2** Images of postoperative complications. A: Scleral impression. B: Hyphema. C: Posterior capsular opacity

**B**

**A**

**C**
