# Supplementary material for: Clinical Efficacy of Ultrasound Cycloplasty for Glaucoma Patients and Pathological Observation on the Ciliary Body in Rabbit Models
Source: J Ophthalmol. 2026 Mar 30;2026:1279697. doi: 10.1155/joph/1279697 (PMC13463128; doi:10.1155/joph/1279697)
Supplement: Supplementary file 3 [file JOPH-2026-1279697-s003.docx]

**Figure.3** Light microscopy. **A** Normal ciliary body tissue (HE×100). **B** The non-pigmented epithelium of the ciliary body disappears, and the arrangement of the pigmented epithelium is disordered (HE×100). **C** Stroma edema (HE×200). **D** No obvious damage to the peripheral retina (HE×40). **E** No obvious damage to the peripheral choroid (HE×100). **F** No obvious damage to the sclera around the target tissue (HE×100).
