# Supplementary material for: Clinical Efficacy of Ultrasound Cycloplasty for Glaucoma Patients and Pathological Observation on the Ciliary Body in Rabbit Models
Source: J Ophthalmol. 2026 Mar 30;2026:1279697. doi: 10.1155/joph/1279697 (PMC13463128; doi:10.1155/joph/1279697)
Supplement: Supplementary file 4 [file JOPH-2026-1279697-s004.docx]

Table 6 Related parameters of the ciliary body

The data of related parameters of the ciliary body are presented as the mean ± SD.

|  | Preoperation | Day 1 | Month 1 | Month 3 |
| --- | --- | --- | --- | --- |
| **Ciliary process**’**height (T value, mm)**﹡ | | | | |
| **3° 'clock** | 1.07±0.24 | 1.12±0.32 | 1.16±0.22 | 1.10±0.37 |
| *t value* | — | -0.516 | -1.898 | -0.138 |
| *P value* | — | 0.615 | 0.094 | 0.896 |
| **6° 'clock** | 0.99±0.21 | 1.24±0.19 | 1.09±0.24 | 0.91±0.15 |
| *t value* | — | -3.381 | -0.596 | 0.443 |
| *P value* | — | 0.005 | 0.568 | 0.199 |
| **9° 'clock** | 1.02±0.15 | 1.09±0.24 | 1.14±0.18 | 1.07±0.26 |
| *t value* | — | -0.456 | -1.473 | -0.443 |
| *P value* | — | 0.429 | 0.179 | 0.673 |
| **12°'clock** | 1.05±0.23 | 1.30±0.25 | 1.05±0.24 | 1.01±0.24 |
| *t value* | — | -2.292 | -0.41 | 0.995 |
| *P value* | — | 0.039 | 0.968 | 0.358 |
| **Ciliary body to iris root attachment points (mm)**﹡ | | | | |
| **3° 'clock** | 0.80±0.15 | 1.03±0.28 | 0.96±0.34 | 0.83±0.30 |
| *t value* | — | -1.969 | -1.96 | -0.033 |
| *P value* | — | 0.085 | 0.069 | 0.975 |
| **6° 'clock** | 0.89±0.20 | 1.05±0.30 | 1.09±0.32 | 1.05±0.43 |
| *t value* | — | -2.07 | -0.995 | -1.274 |
| *P value* | — | 0.056 | 0.349 | 0.25 |
| **9° 'clock** | 0.79±1.67 | 0.96±0.30 | 1.01±0.25 | 0.87±0.171 |
| *t value* | — | -1.511 | -2.007 | -1.621 |
| *P value* | — | 0.165 | 0.08 | 0.144 |
| **12°'clock** | 0.94±0.39 | 1.06±0.32 | 0.89±0.31 | 1.06±0.26 |
| *t value* | — | -0.962 | 0.02 | -1.393 |
| *P value* | — | 0.352 | 0.985 | 0.213 |

Compared with operation, P values lower than 0.05 indicated statistical significance.﹡:A paired sample t test.
