# Supplementary material for: Clinical Efficacy of Ultrasound Cycloplasty for Glaucoma Patients and Pathological Observation on the Ciliary Body in Rabbit Models
Source: J Ophthalmol. 2026 Mar 30;2026:1279697. doi: 10.1155/joph/1279697 (PMC13463128; doi:10.1155/joph/1279697)
Supplement: Supplementary file 5 [file JOPH-2026-1279697-s005.docx]

Table 5 Intraocular pressure(IOP) reduction between previous surgical history group and no previous surgical history group

|  | previous surgical historygroup average  IOP（mmHg） | no previous surgical history group average  IOP（mmHg） | Reduction percentage of previous surgical history group（%） | Reduction percentage of no previous surgical history group（%） |
| --- | --- | --- | --- | --- |
| preoperation | 44.56±12.43 | 45.3±12.39 | — | — |
| Day 1 | 27.33±11.07 | 26.86±8.75 | 38.67% | 40.71% |
| Week 1 | 22.78±9.09 | 20.57±8.44 | 48.88% | 54.59% |
| Month 1 | 16.88±6.65 | 22.14±7.25 | 62.12% | 51.12% |
| Month 3 | 20.75±7.67 | 22.54±6.16 | 53.43% | 40.24% |
| Month 6 | 19.71±7.73 | 23.8±10.69 | 57.77% | 47.46% |
| 1year | 20.38±6.94 | 25.2±11.33 | 54.26% | 44.37% |

The data of IOP are presented as the mean ± SD.
