# Supplementary material for: Clinical Efficacy of Ultrasound Cycloplasty for Glaucoma Patients and Pathological Observation on the Ciliary Body in Rabbit Models
Source: J Ophthalmol. 2026 Mar 30;2026:1279697. doi: 10.1155/joph/1279697 (PMC13463128; doi:10.1155/joph/1279697)
Supplement: Supplementary file 6 [file JOPH-2026-1279697-s006.docx]

Table 4 Intraocular pressure(IOP) reduction between the neovascular glaucoma(NVG） group and the primary angle-closure glaucoma(PACG) group

|  | NVG group average  IOP（mmHg） | PACG group average  IOP（mmHg） | Reduction percentage of NVG group（%） | Reduction percentage of PACG group（%） |
| --- | --- | --- | --- | --- |
| preoperation | 45.38±10.99 | 45.62±13.19 | — | — |
| Day 1 | 26.94±8.75 | 28.46±9.42 | 40.63% | 37.61% |
| Week 1 | 21.63±7.96 | 22.38±8.95 | 52.33% | 50.94% |
| Month 1 | 22.19±7.58 | 22.54±7.33 | 51.10% | 50.59% |
| Month 3 | 23±5.99 | 24.00±7.00 | 49.31% | 47.39% |
| Month 6 | 27.2±12.39 | 20.13±4.19 | 40.0% | 55.87% |
| 1year | 32.33±12.52 | 19.14±3.08 | 28.76% | 58.04% |

The data of IOP are presented as the mean ± SD.
