# Supplementary material for: Clinical Efficacy of Ultrasound Cycloplasty for Glaucoma Patients and Pathological Observation on the Ciliary Body in Rabbit Models
Source: J Ophthalmol. 2026 Mar 30;2026:1279697. doi: 10.1155/joph/1279697 (PMC13463128; doi:10.1155/joph/1279697)
Supplement: Supplementary file 7 [file JOPH-2026-1279697-s007.docx]

Table 3 Success rate in all patients and comparison between primary angle-closure glaucoma(PACG) and neovascular glaucoma(NVG) , surgery history and non surgery history

|  | Day 1 | Week 1 | Month 1 | Month 3 | Month 6 | | 1 year |
| --- | --- | --- | --- | --- | --- | --- | --- |
| All patients | | | | | | | |
| number of successful cases | 39（55） | 36（55） | 32（54） | 30（51） | 21（39） | | 16（33） |
| success rate% | 70.90 | 65.45 | 59.26 | 58.82 | 53.84 | | 48.48 |
| NVG group | | | | | | | |
| number of successful cases | 11（16） | 9（16） | 7（16） | 7（16） | 4（10） | | 1（6） |
| success rate% | 68.75 | 56.25 | 43.75 | 43.75 | 40 | | 16.67 |
| PACG group | | | | | | | |
| number of successful cases | 7（13） | 6（13） | 6（13） | 6（12） | 5（8） | | 5（7） |
| success rate% | 53.84 | 46.15 | 46.15 | 50.0 | 62.5 | | 71.42 |
| Surgery history group | | | | | | | |
| number of successful cases | 14（18） | 14（18） | 13（17） | 11（16） | 8（14） | | 7（13） |
| success rate% | 77.78 | 77.78 | 76.47 | 68.75 | 57.14 | | 53.84 |
| Non-surgery history group | | | | | | | |
| number of successful cases | 25（37） | 22（37） | 19（37） | 19（35） | 13（25） | 9（20） | |
| success rate% | 67.56 | 59.45 | 51.35 | 54.28 | 52.0 | 45.0 | |
