# Supplementary material for: Clinical Efficacy of Ultrasound Cycloplasty for Glaucoma Patients and Pathological Observation on the Ciliary Body in Rabbit Models
Source: J Ophthalmol. 2026 Mar 30;2026:1279697. doi: 10.1155/joph/1279697 (PMC13463128; doi:10.1155/joph/1279697)
Supplement: Supplementary file 8 [file JOPH-2026-1279697-s008.docx]

**Figure.4** Transmission electronmicroscopy. **yellow arrows:** Partially damaged mitochondria autophagy, forming autophagosomes. **black arrows :**Mitochondrial damage, mitochondrial swelling, and dissolution of crists can be seen in the epithelial cells without pigment. **red arrows:**Some golgi bodies are swollen and the lacunae are markedly dilated. **blue arrows:**There is a close connection between pigment epithelial cells and non-pigment epithelial cells, with desmosomes. **purple arrows:**Vascular endothelial cells.
