# Supplementary material for: Clinical Efficacy of Ultrasound Cycloplasty for Glaucoma Patients and Pathological Observation on the Ciliary Body in Rabbit Models
Source: J Ophthalmol. 2026 Mar 30;2026:1279697. doi: 10.1155/joph/1279697 (PMC13463128; doi:10.1155/joph/1279697)
Supplement: Supplementary file 9 [file JOPH-2026-1279697-s009.docx]

Table 1 Patient characteristics and ocular history

| Variable | n(%)/(x±s) |
| --- | --- |
| Age(year) | 45 ± 13 |
| Sex(n) |  |
| Male | 27（49%） |
| Female | 28（51%） |
| Type of glaucoma |  |
| Primary angle-closure glaucoma | 13（24%） |
| Neovascular glaucoma | 16（29%） |
| Other type of secondary glaucoma | 26（47%） |
| Previous glaucoma surgery |  |
| None | 37（67%） |
| Trabeculectomy | 6（11%） |
| Cyclophotocoagulation | 2（4%） |
| Cyclocryotherapy | 1（2%） |
| Trabeculectomy combined with cataract surgery | 9（16%） |
| Preoperative visual acuity(n) |  |
| No light perception | 29（53%） |
| Light perception | 7（13%） |
| Hand motion | 9（16%） |
| Count fingers | 1（2%） |
| 4.0 | 4（7%） |
| 4.5 | 3（5%） |
| 4.6 | 2（4%） |
| Axial lenghth(mm) | 23.57±2.09 |
| White to white(mm) | 11.22±0.63 |

The data of Age, Axial lenghth and White to white are presented as the mean ± SD.
