# Supplementary material for: Clinical Efficacy of Ultrasound Cycloplasty for Glaucoma Patients and Pathological Observation on the Ciliary Body in Rabbit Models
Source: J Ophthalmol. 2026 Mar 30;2026:1279697. doi: 10.1155/joph/1279697 (PMC13463128; doi:10.1155/joph/1279697)
Supplement: Supplementary file 10 [file JOPH-2026-1279697-s010.docx]

Table 2 Intraocular Pressure(IOP), Antiglaucoma Medications and the Visual Analogue Scale (VAS)scores

|  | Day 1 | Week 1 | Month 1 | Month 3 | Month 6 | 1 year |
| --- | --- | --- | --- | --- | --- | --- |
| Number (n) | 55 | 55 | 54 | 51 | 39 | 33 |
| **IOP(mmHg)**﹡ | | | | | | |
| preoperation | 45.05±12.29 | 45.05±12.29 | 44.94±12.38 | 44.94±12.57 | 44.44±12.44 | 43.76±13.34 |
| postoperation | 27.02±9.47 | 21.29±8.64 | 20.48±7.43 | 21.98±6.65 | 22.33±9.83 | 23.3±10.0 |
| *t* value | 10.577 | 13.297 | 13.8 | 11.632 | 8.863 | 8.344 |
| *P* value | ＜0.001 | ＜0.001 | ＜0.001 | ＜0.001 | ＜0.001 | ＜0.001 |
| **Antiglaucoma medications**^﹟^ | | | | | | |
| preoperation | 2(2,3) | 2(2,3) | 2(2,3) | 2(2,3) | 2(2,3) | 2(2,3) |
| postoperation | 1(1,2) | 2(0,2) | 1.5(0,2) | 2 (1,2) | 1(0,2) | 1(1,2) |
| *Z* value | -5.946 | -5.481 | -5.672 | -5.218 | -5.29 | -4.997 |
| *P* value | ＜0.001 | ＜0.001 | ＜0.001 | ＜0.001 | ＜0.001 | ＜0.001 |
| **VAS scores**^﹟^ | | | | | | |
| preoperation | 5(4,6) | 5(4,6) | 5(4,6) | 5(4,6) | 5(4,6) | 5(4,6) |
| postoperation | 3 (2,3) | 1 (0,2) | 1(0,2) | 1 (0,1) | 1(0,2) | 1 (1,2) |
| *Z* value | -6.02 | -6.424 | -6.429 | -6.257 | -5.298 | -5.006 |
| *P* value | ＜0.001 | ＜0.001 | ＜0.001 | ＜0.001 | ＜0.001 | ＜0.001 |

The data of IOP is presented as the mean ± SD.The data on the number of antiglaucoma medications and the VAS scores are expressed as M (Q1, Q3).Compared with operation, P values lower than 0.05 indicated statistical significance.﹡:A paired sample t test ;^﹟^:the Wilcoxon rank sum test.
